# Combination treatment with intravesical interferon-alpha gene therapy and an oral pan-ErbB receptor family blocker improves survival in mice with bladder cancer

**DOI:** 10.3389/fmolb.2026.1804161

**Published:** 2026-08-10

**Authors:** Akshay Sood, Alberto Martini, Paulo Genitiano, Mikael Rauf, Ganiraju Manyam, Jan K. Rudzinski, Come Tholomier, Roberto Contieri, I-Ling Lee, Nigel R. Parker, Seppo Yla-Herttuala, David J. McConkey, Colin P. N. Dinney, Sharada Mokkapati

**Affiliations:** 1 Department of Urology and Bioinformatics and Computational Biology, The University of Texas MD Anderson Cancer Center, Houston, TX, United States; 2 Department of Urology, The James Cancer Hospital and Solove Research Institute, The Ohio State University Wexner Medical Center, Columbus, OH, United States; 3 A.I. Virtanen Institute for Molecular Sciences, University of Eastern Finland, Kuopio, Finland; 4 Department of Urology, University of Rochester Medical Center, Rochester, NY, United States

**Keywords:** afatinib, bladder cancer, epidermal growth factor receptor, gene therapy, mice

## Abstract

**Introduction:**

Intravesical interferon-alpha (IFNα) gene therapy is approved by the FDA for BCG-unresponsive non-muscle invasive bladder cancer (NMIBC). Identifying resistance mechanisms and deploying targeted combination treatment strategies is a rational approach to improving treatment responses. We identified the ErbB pathway as a resistance mechanism to IFNα gene therapy, and we hypothesized that combination treatment with an ErbB pathway blocker and IFN-α could improve outcomes in resistant tumors.

**Methods:**

Murine bladder cancer cells were treated *in vitro* with lentiviral IFNα (LV-IFNα) gene therapy, with/without afatinib (Afa), a pan-ErbB inhibitor, and cell viability and migration assays were performed. *In vivo* studies were conducted in a syngeneic MB49 orthotopic murine bladder cancer model, and mice were randomized into five treatment groups and treated with single treatments: Ctrl (vehicle), LV-Ctrl, LV-IFNα, and combination treatments with LV-Ctrl/Afa or LV-IFNα/Afa.

**Results:**

Combination therapy with LV-IFNα/Afa significantly reduced MB49 cell viability *in vitro* compared to all other treatment conditions. This additive effect on cell viability appeared to be driven by a combination of early-cytostatic and late-cytolytic effects. The combination treatment also markedly inhibited cell migration. Finally, the *in vivo* studies demonstrated improved overall survival (OS) with LV-IFNα/Afa (median OS was 49 days in the LV-IFNα/Afa group vs. 15, 14, 29, and 26 days in Ctrl, LV-Ctrl, LV-IFNα, and LV-Ctrl/Afa groups, respectively; log-rank *p* < 0.001).

**Conclusion:**

Our findings suggest that the ErbB pathway may serve as a clinically actionable resistance mechanism to intravesical IFNα gene therapy and, when targeted concurrently, it may improve treatment efficacy.

## Introduction

The ErbB family of receptors plays a crucial role in several key cellular homeostatic functions, including cell growth, proliferation, differentiation, apoptosis, and motility, and, when dysregulated, can promote tumorigenesis (Hynes and MacDonald, 2009; Hynes and Lane, 2005). The recently concluded The Cancer Genome Atlas (TCGA) project, which profiled 412 high-grade bladder tumors, found that the *EGFR*, *ERBB2*, and *ERBB3* genes were among the top 10 most frequently altered genes in patients with urothelial carcinoma of the bladder (Robertson et al., 2017). Notably, expression of ErbB pathway receptors is associated with disease stage and survival in patients with bladder cancer (Kassouf et al., 2008). EGFR has been identified as a potential therapeutic target in a subset of muscle-invasive bladder cancers, particularly the basal-like subtype (Rebouissou et al., 2014), and high ERBB2 expression correlated with reduced recurrence-free survival in patients with NMIBC (Sikic et al., 2022). ERBB2 mutations have also been shown to promote recurrence and metastasis through HIF-1α phosphorylation (Wang et al., 2026).

Nadofaragene firadenovec is an intravesically administered, replication-deficient, adenoviral vector-delivered interferon-alpha (IFNα) gene therapy that has demonstrated promising results in the treatment of patients with BCG-unresponsive non-muscle invasive bladder cancer (NMIBC) (Shore et al., 2017; Boorjian et al., 2021; Narayan et al., 2024) and was granted FDA approval for this indication in 2022. Of the 151 patients studied in a phase-III clinical trial, 59.6% achieved complete pathologic response at the initial 3-month assessment, with 51.1% maintaining a durable response at 12 months (Boorjian et al., 2021). Although the results of this trial have been encouraging, ongoing research in our laboratory is focused on further enhancing treatment efficacy and durability. Strategies have included biomarker-driven candidate selection (Mitra et al., 2022), development of innovative drug delivery vectors (Mokkapati et al., 2022), and identification of novel resistance mechanisms for targeted combination therapies. The current study investigates the last strategy, whereby we identified the ErbB pathway as a significant resistance mechanism to intravesical IFNα gene therapy (Mokkapati et al., 2022) and sought to test the combined effect of intravesical IFNα gene therapy and an oral pan-ErbB receptor blocker on survival and tumor progression outcomes in the MB49 syngeneic orthotopic murine model of bladder cancer *in vivo* and in MB49, BBN975, and UPPL1541 murine bladder cancer cell lines *in vitro*.

## Materials and methods

### Cell lines

For this study, we tested the efficacy of combination treatment with lentiviral IFNα (LV-IFNα) and a pan-ERBB inhibitor using three syngeneic cell lines previously described and evaluated in our group (Mokkapati et al., 2022). Cells were cultured in MEM (Corning 10-010-CV; MB49) or DMEM (Corning, 10-017-CV) supplemented with 10% FBS (Sigma, F0926; heat-inactivated), penicillin/streptomycin (Gibco, 15070-063), sodium pyruvate (Gibco, 11360-070), and non-essential amino acids (Gibco, 11140-050). We tested these three cell lines as they encompass the molecular and physical phenotypes encountered clinically in bladder cancer (Kobayashi et al., 2015; Saito et al., 2018).

### Viral vectors

Lentiviral vectors expressing IFNα (LV-IFNα) and control vectors were manufactured at the University of Eastern Finland and previously tested in our laboratory (Mokkapati et al., 2022). For viral transduction, cells were seeded at a density of 1000 cells per well in a 96-well plate or at varying densities in a 6-well culture dish depending on the growth characteristics and size of the cells (MB49 at 50,000; BBN975 at 75,000; and UPPL1541 at 75,000 cells/well). After overnight attachment, cells were treated with either LV-Ctrl (MOI 2) or LV-IFNα (MOI 2), either alone or in combination with afatinib at IC_50_ concentrations: MB49, 0.10 μM; BBN975, 0.05 μM; and UPPL1541, 0.15 μM. Control cells were maintained in media alone. For all viral transductions, media was supplemented with polybrene (4 μg/mL).

### Cell viability and migration assays

Cell viability was measured by seeding cells in 96-well cell culture dish and treated with the agents as described above. A 3-day cell viability was measured using CellTiter Glow Luminescent Cell viability Assay (Promega, G7573) according to the manufacturer’s instructions. For measuring apoptosis, cells were seeded at a density of 30,000 cells per well in a 6-well cell culture dish and treated with the agents as described above. eBioscienceTM Annexin V Apoptosis Detection Kit (ThermoFisher Scientific, 88-8007-74) was used to determine percentage of cell death at 24 h and 72 h post treatment using Flow cytometry. Nuclear isolation medium4,6-diamidino-2-phenylindole dihydrochloride (NIM-DAPI) was used to measure cell cycle changes following treatment at 24 h and 72 h post treatment.

For cell invasion assay, trypsinized cells were suspended in serum-free media (500 μL) and seeded in the upper chamber of a 24-well Matrigel invasion chamber (Corning, 3544080) at a density of 150,000 cells/well, with media containing 10% FBS in the lower chamber serving as a chemoattractant for cell migration. After 36-h, cells that invaded through the 8 μm pore were fixed in 4% paraformaldehyde and stained with crystal violet. Results were quantified using a 4-quadrant approach (×10 magnification) using NIH ImageJ software (Schneider et al., 2012). All experiments had at least three biological replicates.

### *In vivo* syngeneic orthotopic murine model

Animal experiments conducted in this study followed the Institutional Animal Care and Use Committee (IACUC) guidelines at MD Anderson Cancer Center. Intravesical MB49 tumors were established in female C57BL/6 mice as described previously (Mokkapati et al., 2022).

Tumor burden was assessed on day 4 post-instillation using the IVIS spectrum *in vivo* imaging system equipped with Living Image Software (PerkinElmer). Mice were randomized into five treatment groups (n = 10 animals/group), stratified by tumor uptake to ensure equal baseline tumor burden distribution. Treatment and control arms consisted of saline control (Ctrl), LV-Ctrl (3 × 10^7^ virus particles in 100 μL PBS x one-time 40-min instillation), afatinib monotherapy (5 mg/kg daily oral gavage with rest on weekends), LV-IFNα monotherapy (3 × 10^7^ virus particles in 100 μL PBS x one-time 40-min instillation), and the experimental LV-IFNα + afatinib combination therapy. Tumor growth was monitored using luciferase imaging once weekly. Animals were monitored daily, and mice with body weight reduction (>20%) accompanied by significant lethargy or hematuria were deemed moribund and euthanized as per IACUC protocol. All animals were euthanized 9 weeks from treatment start by CO_2_ inhalation (4-7 L/min), followed by cervical dislocation. Laparotomy was performed, and tissue specimens were collected immediately following euthanasia. Mice were weighed at necropsy, and bladders were removed and weighed. The *in vivo* experiments were conducted thrice.

### Gene expression profiling

Primary analysis of RNA-seq data was performed using the GSE205493 dataset as a reference for identifying changes in the ErbB pathway (Mokkapati et al., 2022). For RNA-seq analysis, 30–100 million reads per sample were analyzed. Differential expression analysis was performed using t-tests, and the false discovery rate (FDR) was estimated using the beta-uniform mixture method (Pounds and Morris, 2003).

### Reverse phase protein array analysis

Cells were seeded in 6-well plates and treated as described above; protein lysates were prepared from cells collected after 24 h of exposure to the agents and subjected to reverse-phase protein array (RPPA) analysis at the MD Anderson RPPA Core Facility. The methods used at the core facility have been validated in previous publications (Tibes et al., 2006). Serially diluted protein lysates were printed onto nitrocellulose-coated slides and probed with >400 antibodies. Relative protein expression was determined for each sample to compare protein expression after treatment. The normalized RPPA matrix and details of the antibodies are provided in Supplementary Table S3.

### Statistical methods

GraphPad Prism 10 Software was used for statistical analyses. Multiple-group comparisons were performed using two-way ANOVA or Kruskal–Wallis tests for continuous data. The chi-square test was used for categorical data. Survival analyses were performed using the Kaplan–Meier method with the log-rank test. A *p-*value < 0.05 was considered statistically significant.

## Results

### ErbB pathway activation acts a resistance mechanism to LV-IFNα therapy

**FIGURE 1:**
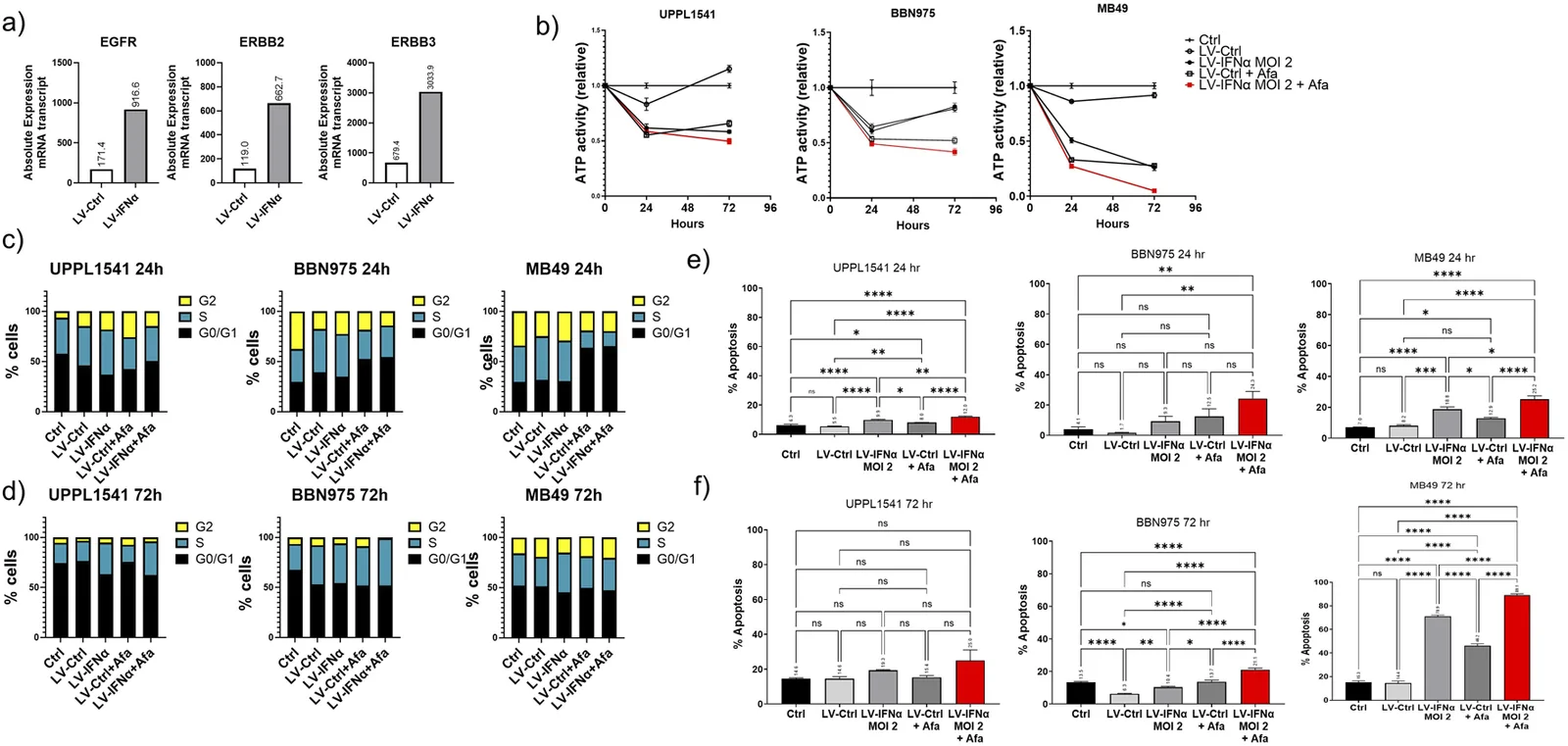
Cell cycle and viability analysis of murine bladder cancer cell lines treated with LV-IFNα and the pan-ERBB inhibitor afatinib. **(a)** RNA-seq analysis demonstrating EGFR, ERBB2, and ERBB3 upregulation in *in vivo* MB49 end-tumors following LV-IFNα treatment. **(b)** *In vitro* cell viability assay through 96 h. **(c,d)** *In vitro* cell cycle (G0/G1 arrest, S phase, and G2 phase) analysis at 24 h and 72 h; **(e,f)** apoptosis assay at 24 h and 72 h in cell lines treated *in vitro*. *p*-values: not significant (n.s.); *p* > 0.05 (n.s.); *p* < 0.05 (*); *p* < 0.01 (**); *p* < 0.0001 (****).

We performed post hoc end-tumor RNA-seq analysis of MB49 murine tumors treated with LV-IFNα and LV-Ctrl (as part of a prior study) (Mokkapati et al., 2022) and demonstrated a 4- to 6-fold upregulation of the ErbB pathway in treatment-resistant end-tumors. Differential gene expression identified 1,671 genes (885 upregulated and 786 downregulated) when comparing LV-Ctrl with LV-IFNα. All genes are shown in Supplementary Table S1, with ERBB pathway genes specifically highlighted. Pathway analysis also identified ERBB-related pathways as being significantly enriched in the LV-IFNα group (Supplementary Figure S1). A complete list of differentially expressed genes for these pathways is provided (Supplementary Table S2). Specifically, the absolute mRNA expression levels for EGFR, ERBB2, and ERBB3 in these treatment arms were 919.6 vs. 171.4, 662.7 vs. 119.0, and 3,033.9 vs. 679.4, respectively (*p* < 0.001) (Figure 1a). Based on these observations and prior studies demonstrating a high prevalence of ErbB mutations in bladder cancer (Robertson et al., 2017; Chen et al., 2021), we aimed to evaluate the *in vitro* and *in vivo* efficacy of LV-IFNα + afatinib combination therapy. To clinically correlate our findings, we evaluated mRNA expression of EGFR, ERBB2, ERBB3, and ERBB4 in pre- and post-treatment tumors of eight patients treated with nadofaragene firadenovec (phase 2 clinical trial). Of the 8 samples, EGFR was upregulated in 1/8 post-treatment samples, ERBB2 in 2/8, ERBB3 in 3/8, and ERBB4 in 4/8 samples. Overall, 75% (6/8) of patients exhibited upregulation of at least one of the four proteins, and 25% (2/8) exhibited upregulation of three of the four genes (Supplementary Figure S2).

### LV-IFNα + afatinib combination therapy reduces cell viability and exerts additive effects on apoptosis in the MB49 murine cell line

*In vitro* testing showed that combination therapy significantly decreased the viability of MB49 cells compared to all other treatment conditions. The mean relative ATPase activity at 72 h was 4% for the combination treatment, contrasting with 100%, 96%, 26%, and 28% for the Ctrl, LV-Ctrl, LV-IFNα, and afatinib arms, respectively (*p* < 0.001) (Figure 1b). These findings were not observed in the UPPL1541 and BBN975 cell lines where combination treatment did not significantly affect cell viability compared to monotherapy (see Figure 1b). This additive effect on cell viability in MB49 cells appeared to be driven by a combination of early cytostatic and late cytolytic effects. At the 24 h time point, 65.3% of cells in the combination treatment arm were in G0/G1 arrest compared to 29.7% in the Ctrl group. This cytostatic effect was likely facilitated by the rapid onset of afatinib’s anti-ErbB activity, as evidenced by cell-cycle arrest in the afatinib monotherapy arm (Figure 1c). At the 72 h time point, the degree of cytostasis was similar across all treatment arms (Figure 1d). However, the rate of apoptosis was significantly increased in the LV-IFNα/Afa group compared to the LV-IFNα group, both at 24 h (25.2% vs. 18.8, *p* < 0.05) and at 72 h (70.9% and 46.2% at 72 h, *p* < 0.001), respectively (Figures 1e,f).

### LV-IFNα + afatinib combination therapy reduces cell migration in all three murine cell lines

**FIGURE 2:**
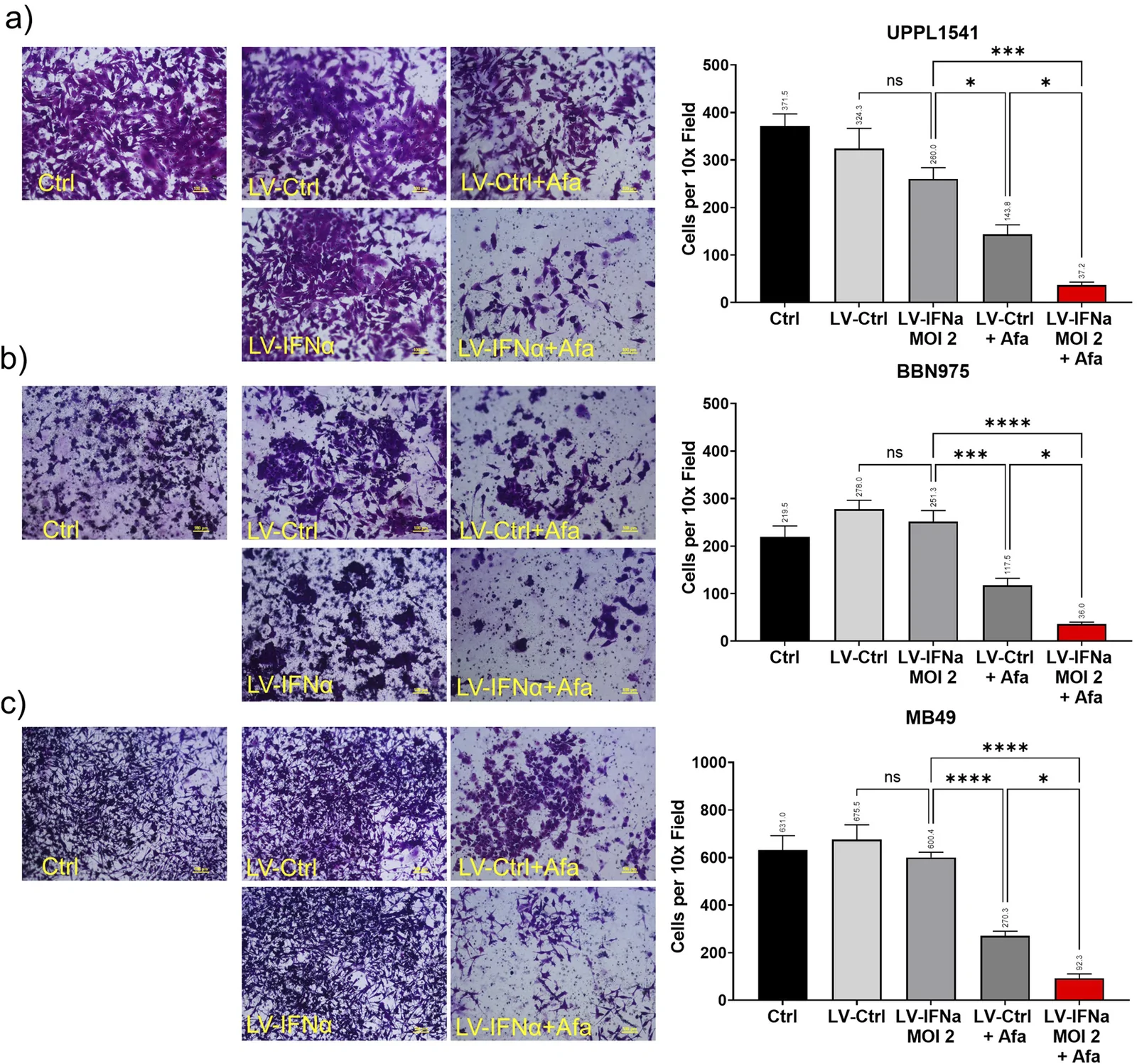
*In vitro* Boyden chamber cell invasion assay and quantification at 36 h in bladder cancer cell lines treated with LV-IFNα and the pan-ERBB inhibitor afatinib. **(a)** Representative microscopic images and quantification of the indicated treatment groups in the cell lines UPPL1541 and **(b)** BBN975 and **(c)** MB49 cells. *p* > 0.05 (n.s.); *p* < 0.05 (*); *p* < 0.01 (**); *p* < 0.0001 (****).

The combination treatment markedly inhibited cell migration and invasion across all three cell lines. The mean number of migrated MB49 cells/10x Boyden chamber assay was 92.3 for the LV-IFNα+Afa group and 631.0, 675.5, 600.4, and 270.3 for Ctrl, LV-Ctrl, LV-IFNα, and LV-Ctrl + Afa groups, respectively (*p* < 0.001), as measured at the 36 h mark (Figure 2c). Similar results were observed for the UPPL1541 and BBN975 cell lines (Figure 2a,b).

### The efficacy of combination therapy correlates with the cell line’s dependence on the ErbB pathway as an escape mechanism following LV-IFNα treatment

**FIGURE 3:**
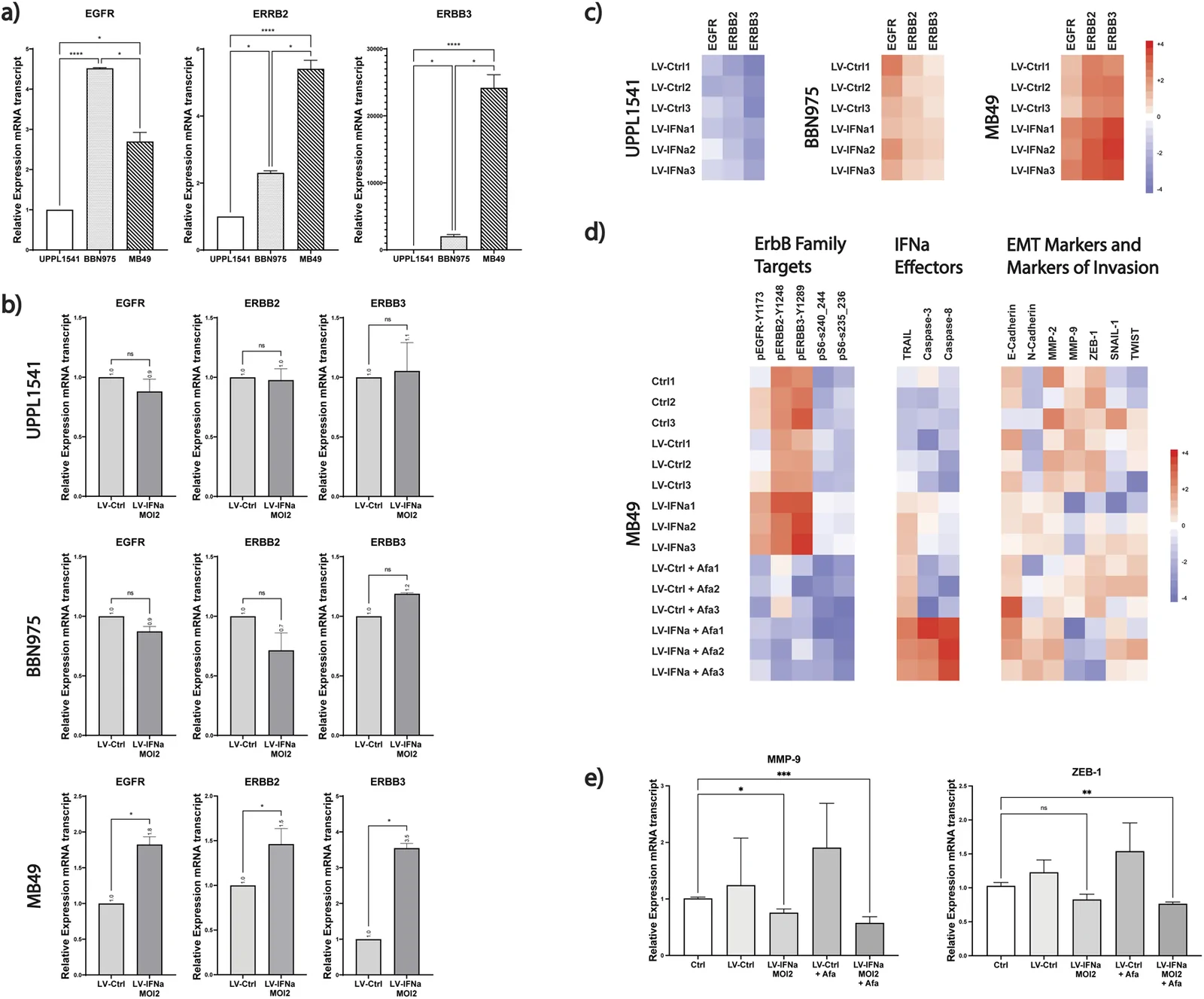
Identification of potential mechanism of action *in vitro*. **(a)** Baseline mRNA expression of EGFR, ERBB2, and ERBB3 across the three cell lines. **(b)** Effect of LV-IFNα treatment on mRNA expression levels of EGFR, ERBB2, and ERBB3 across the three cell lines. **(c)** RPPA analysis of the effect of LV-IFNα treatment on protein-level expression of EGFR, ERBB2, and ERBB3 across the 3 cell lines; **(d)** RPPA analysis on the effect of combination treatment on ErbB, IFNα, and EMT pathways in the MB49 cell line. **(e)** RT-qPCR analysis demonstrating the suppression of MMP-9 and ZEB-1 with combination treatment in the MB49 cell line. *p* > 0.05 (n.s.); *p* < 0.05 (*); *p* < 0.01 (**); *p* < 0.0001 (****).

The baseline expression levels of EGFR, ERBB2, and ERBB3 mRNA transcripts in the MB49, BBN975, and UPPL1541 cell lines correlated with the observed treatment efficacy, as measured by apoptosis following afatinib treatment and combination therapy. For example, the UPPL1541 cell line, which displayed low baseline levels of ErbB receptors (Figure 3a), showed minimal responsiveness to afatinib. In contrast, both BBN975 and MB49 cell lines responded well. This is in line with previous work on anti-ErbB therapy and ErbB expression in solid human tumors (Baselga et al., 2002). The success of combination therapy, however, appeared to be dependent on the extent to which each cell line relied on the ErbB pathway as a compensatory mechanism following IFNα therapy. As illustrated in Figure 3b, MB49 cells exhibited an increase in the expression of EGFR (1.8-fold), ERBB2 (1.5-fold), and ERBB3 (3.5-fold) mRNA transcripts post-LV-IFNα treatment, while the other cell lines did not. These observations were confirmed on RPPA proteomic analysis (see Figure 3c). Additional proteomic analysis of the MB49 cell line further showed that LV-IFNα exposure also triggered an activation of the ErbB pathway, as evidenced by enhanced phosphorylation of the EGFR-pY1173, HER2-pY1248, and HER3-pY1289 receptors and the downstream effectors (pS6-240_244 and pS6-235_236, as shown in Figure 3d, left panel). We tested the efficacy of the combination therapy in the human bladder cancer cell line UC6 and found a 2-fold increase in EGFR expression and a reduction in IC_50_ from 18.3 nM when treated with afatinib monotherapy to 3.301 nM when treated with the combination therapy afatinib and 1000 MOI Ad-IFNα (Supplementary Figures 3a,b). Afatinib therapy effectively inhibited this activation (Figure 3d, left panel). Finally, afatinib synergistically enhanced the response to LV-IFNα therapy, as indicated by increased levels of TRAIL, caspase-3, and caspase-8, key mediators of IFNα response (Figure 3d, middle panel). These mechanistic insights align with the distinct tumor cell growth responses observed across the three cell lines (shown in Figure 1b), supporting afatinib’s selective mechanism of action in cells that utilize the ErbB pathway as an escape mechanism (Tang et al., 2015).

### Suppression of cell invasion is likely mediated by the inhibition of MMP-9, ZEB-1, and membranous EGFR and does not appear to parallel the cytostatic effects of afatinib

Preliminary findings from protein and RNA-seq analyses indicated that the suppression of MMP-9 (Sier et al., 2000) and ZEB-1 (Caramel et al., 2018) likely played a role in the diminished invasive potential of MB49 cells treated with the combination therapy (Figures 3d, right panel; Figures 3e). Other factors, such as E-cadherin, N-cadherin, MMP-2, SNAIL-1, and TWIST, were also evaluated and did not appear to be altered by the combination therapy. MMP-9 (but not ZEB-1) was also suppressed in the UPPL1541 and BBN975 cell lines [data not shown]. However, MMP-9 and ZEB-1 likely act in concert with other mechanisms that reduce invasive potential as cells treated with afatinib monotherapy also demonstrated a significant reduction in invasion compared to the control groups (Figure 2), despite showing no differences in expression levels of MMP-9 and ZEB-1 (Figure 3d, right panel). Previous research from our laboratory has demonstrated that a significant pool of activated EGFR is localized to dynamic F-actin-containing structures located at focal sites of cellular motility (such as membrane ruffles) and is inhibited by ErbB inhibitors in a cell type-agnostic manner (Kassouf et al., 2005). Taken together, these findings suggest that the additive inhibitory effect of combination treatment on motility observed in the afatinib + LV-IFNα arm is likely mediated by MMP-9/ZEB-1 inhibition and ErbB receptor dephosphorylation.

### LV-IFNα + afatinib combination therapy reduces tumor burden and improves survival

**FIGURE 4:**
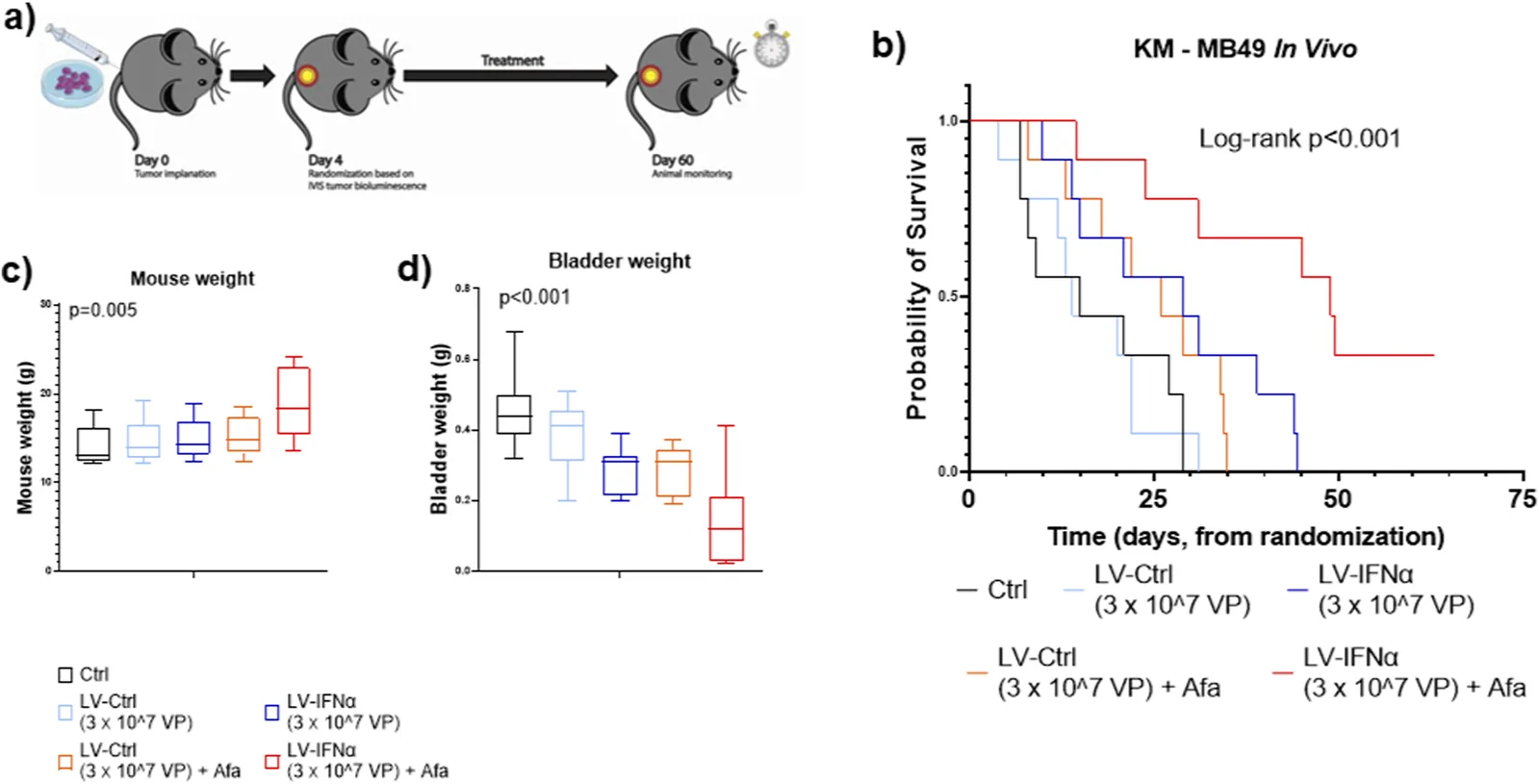
Efficacy of combination treatment in MB49 intravesical model. **(a)** Schematic of *in vivo* experiments in the MB49 syngeneic orthotopic bladder cancer model. **(b)** Kaplan–Meier plot of overall survival in the five treatment arms. **(c)** Total body weight at study end or euthanasia [a surrogate measure of overall health status]. **(d)** Bladder weight at study end or euthanasia [a surrogate measure of local tumor growth].

The schematic of the *in vivo* study is shown in Figure 4a. Figure 4b demonstrates improved OS with combination therapy. The median OS was 49 days in the combination group vs. 15, 14, 29, and 26 days in the Ctrl, LV-Ctrl, LV-IFNα, and afatinib groups, respectively (log-rank *p* < 0.001) (Figure 4b). The combination therapy group also had significantly higher mouse weights (a surrogate measure of overall health status) compared to the other groups (Figure 4c), while the bladder weights (a surrogate measure of local tumor burden) were significantly lower in the combination therapy group (Figures 4d). No mice in the combination therapy group died from treatment-related toxicity.

## Discussion

BCG-unresponsive NMIBC is an inherently resistant cancer. Radical cystectomy remains the standard-of-care but is associated with significant morbidity and long-term quality of life considerations. Understandably, many patients are reluctant to proceed with radical cystectomy, so the identification of well-tolerated alternatives to radical cystectomy that provide durable clinical responses has become a major research focus for NMIBC.

Over the past several decades, numerous novel drugs have been evaluated for this indication, including nadofaragene firadenovec (Boorjian et al., 2021), cretostimogene grenadenorepvec (CG0070) (Packiam et al., 2018), pembrolizumab (Balar et al., 2017; Necchi et al., 2024), gemcitabine/docetaxel (Steinberg et al., 2015; Chevuru et al., 2023), and N-803/BCG (Chamie et al., 2023), among multiple others in trials currently (Hannouneh et al., 2023). These drugs have demonstrated variable success, but those rigorously evaluated in prospective trials typically exhibited an 18-month complete response rate ranging from 20% to 30%. The high rate of early post-treatment resistance highlights the urgent need for strategies to improve the durability and efficacy of current therapies. The results of our study underscore the potential of combining intravesical IFNα gene therapy with afatinib, an FDA-approved oral pan-ErbB receptor blocker, for the treatment of patients with BCG-unresponsive NMIBC in whom ERBB pathway activation is induced by intravesical IFNα gene therapy. Our results show that this combination therapy significantly inhibits tumor growth (by >95%), retards cellular invasion (by >80%) *in vitro,* and improves survival *in vivo* (by >300%) in a syngeneic orthotopic mouse model, highlighting its potential as a viable treatment strategy. Specifically, the efficacy of afatinib in this combination underscores the critical role of the ErbB signaling pathway in bladder cancer pathogenesis, consistent with findings from the TCGA project, which identified EGFR, HER2, and HER3 among the most frequently altered genes in high-grade bladder tumors. Our study also aligns with recent pharmacological advances in metastatic bladder cancer, which have demonstrated the potential of targeting the ErbB pathway in bladder malignancy via biomarker profiling of patients and specifically using either dual anti-HER-2 therapies or novel antibody–drug conjugate (ADC) agents such as disitamab vedotin or trastuzumab duocarmazine (Patelli et al., 2022). While these drugs are currently being tested in refractory metastatic urothelial cancers, the same principles can be transferred to treatment-resistant NMIBC. Additionally, our work complements the recently opened ABLE-22 trial (NCT06545955), which aims to explore the concurrent use of nadofaragene firadenovec with pembrolizumab or gemcitabine/docetaxel based on similar pre-clinical work in murine models. These studies share the same goal of improving the efficacy of Ad-IFN gene therapy for patients with BCG-unresponsive NMIBC.

Our mechanistic findings align with the known mechanisms of anti-ErbB and IFNα therapies (Hynes and MacDonald, 2009; Hynes and Lane, 2005; Schneider et al., 2014; Papageorgiou et al., 2007), demonstrating activation (or suppression) of well-characterized downstream pathways that explain our phenotypic observations. Preliminary findings from our work also highlight the involvement of EMT pathways in bladder cancer progression, including MMPs and ZEB-1 (Caramel et al., 2018). While MMP-9 was downregulated only in the MB49 cell line, ZEB-1 was downregulated in all three murine cell lines following combination treatment. ZEB-1, which mediates E-cadherin repression (similar to many other EMT-associated molecules), is a transcriptional regulator that has been implicated in the progression of uterine and colorectal cancers (Spoelstra et al., 2006; Spaderna et al., 2006). One limitation of our study is that we were unable to confirm downregulation of ErbB pathway proteins *in vivo* in our murine tumors.

Our preliminary results identified ERBB pathway upregulation in resistant tumors from patients treated with nadofaragene. This observation requires validation in patients enrolled in phase III clinical trials. While the findings from the current study should be regarded as preliminary, they provide a rationale for formally evaluating the combined use of IFNα gene therapy and afatinib in BCG-unresponsive NMIBC in a future clinical trial. This work builds upon our strategy to enhance intravesical IFNα gene therapy through the identification of biomarkers that predict sensitivity and resistance and the development of novel combination approaches that target resistance mechanisms.

## Data Availability

The raw data supporting the conclusions of this article will be made available by the authors without undue reservation.

## Supplementary material

SUPPLEMENTARY FIGURE S1 
Ingenuity pathway analysis (IPA) of pathways altered between LV-Ctrl and LV-IFNα treatment. RNA-seq data were analyzed using IPA, and differentially expressed pathways, including three EGFR-related pathways (ERBB, ERBB2, and ERBB4), are shown (-log (*p*-value)>2.2; absolute z-score >2.3). Z-scores are indicated above the bars.

SUPPLEMENTARY FIGURE S2
Analysis of RNA-seq data showing differences in the expression of ErbB pathway genes. Normalized expressions levels were compared between pre- and post-treatment samples from eight patients treated with nadofaragene, **(a)** EGFR, **(b)** ERBB2, **(c)** ERBB3, and **(d)** ERBB4.

SUPPLEMENTARY FIGURE S3
Combination treatment with afatinib and Ad-IFNα in UC6 cells. **(a)** q-PCR analysis of EGFR expression in UC6 cells treated with Ad-IFNα. **(b)** Dose-response curves for afatinib monotherapy and combination therapy with afatinib and Ad-IFNα (1000 MOI).
